# Important molecular genetic markers of colorectal cancer

**DOI:** 10.18632/oncotarget.9796

**Published:** 2016-06-02

**Authors:** Anna V. Kudryavtseva, Anastasia V. Lipatova, Andrew R. Zaretsky, Alexey A. Moskalev, Maria S. Fedorova, Anastasiya S. Rasskazova, Galina A. Shibukhova, Anastasiya V. Snezhkina, Andrey D. Kaprin, Boris Y. Alekseev, Alexey A. Dmitriev, George S. Krasnov

**Affiliations:** ^1^ Engelhardt Institute of Molecular Biology, Russian Academy of Sciences, Moscow, Russia; ^2^ National Medical Research Radiological Centre, Ministry of Healthcare of the Russian Federation, Moscow, Russia; ^3^ Shemyakin-Ovchinnikov Institute of Bioorganic Chemistry, Russian Academy of Sciences, Moscow, Russia; ^4^ Orekhovich Institute of Biomedical Chemistry, Russian Academy of Medical Sciences, Moscow, Russia

**Keywords:** colorectal cancer, molecular markers, chromosomal instability, microsatellite instability, CpG island methylator phenotype

## Abstract

Colorectal cancer (CRC) ranks third in the incidences of cancer morbidity and mortality worldwide. CRC is rather heterogeneous with regard to molecular genetic characteristics and pathogenic pathways. A wide spectrum of biomarkers is used for molecular subtype determination, prognosis, and estimation of sensitivity to different drugs in practice. These biomarkers can include germline and somatic mutations, chromosomal aberrations, genomic abnormalities, gene expression alterations at mRNA or protein level and changes in DNA methylation status. In the present review we discuss the most important and well-studied CRC biomarkers, and their potential clinical significance and current approaches to molecular classification of colorectal tumors.

## INTRODUCTION

Colorectal cancer (CRC) is one of the most common cancers in the world [1]. Despite the fact that CRC is histologically homogeneous, each tumor has a unique molecular profile, which is characterized by various genetic and epigenetic changes. Several molecular genetic markers, which are currently used for CRC diagnosis, prognosis and treatment assignment, have been identified. Numerous molecular genetic studies of CRC have revealed many genes that are characterized by high frequency of mutations *(KRAS*, *NRAS*, *BRAF*, *PIK3CA*, *APC*, *TP53*, *SMAD2*, *SMAD4*, *ARID1A*, *SOX9*, *FAM123B*/*WTX*, and *FBXW7*), copy number alterations (*ERBB2* and *IGF2*), methylation status changes (*MLH1*), impaired expression at the mRNA or protein level, and translocations (*NAV2*/*TCF7L1*) [2, 3]. Alteration in many genes have predictive value for assessing prognosis and sensitivity to various drugs. However, the data on their clinical significance are often scattered, ambiguous and even conflicting. In this review, we summarized data on the most important widely used and prospective markers of CRC prognosis, prediction of the response to therapy and evaluated their significance.

Several approaches of CRC classification based on genomic, transcriptomic and proteomic profiling are known. Multiple studies of CRC molecular features suggest the existence of several major molecular subtypes of colorectal cancer, which differ in the mechanisms of their development, course of the disease, and response to various drugs [4-6]. However, exhaustive classification of CRC into molecular subtypes is restricted by a large number of individual tumor features that do not fit the overall picture. The second aim of this review is to describe known approached of CRC classification based on genome-wide features (methylation, expression profiling), alterations in individual genes (driver mutations, deletions) and pathogenic pathways.

## CHROMOSOMAL INSTABILITY OVERVIEW OF PATHWAYS INVOLVED IN COLORECTAL CARCINOGENESIS

Tumors with chromosomal instability (CIN) are often thought of as a separate biological entity. CIN is the most common feature (65-85% tumors) of colon adenocarcinomas compared to others (MSI, CIMP) [4, 7]. CIN-positive tumors are characterized with severely increased frequency of duplications or losses of chromosome regions or entire chromosomes. Despite the fact that a number of methods are currently available in clinical laboratories (e.g., FISH, karyotyping, comparative genomic hybridization (CGH), including microarray-based CGH), it is not always possible to carry out the differential diagnosis of CIN phenotype in routine practice. In many ways, this is related to the variety of chromosomal abnormalities following CIN and the difficulty of developing clear quantitative criteria for diagnosis. CIN tumors are conventionally divided into two subgroups depending on the frequency of chromosomal aberrations: CIN-high (CIN-H) and CIN-low (CIN-L) tumors. CIN tumors are characterized with mutations in various tumor suppressor genes: *APC* (up to 85%), *TP53* (40-50%), *SMAD2/4* (10-20%), and *DCC* (5%), and proto-oncogenes: *KRAS* (30-50%), *CTNNB1* (5-15%), and *PIK3CA* (20%) [4].

The development of most colorectal tumors is caused by dysregulation of several signal transduction pathways - Wnt, TGFβ/BMP, RTK/Ras, PI3K/Akt. Activation of canonical Wnt pathway contributes to the onset and progression of more than 90% colorectal adenocarcinomas and adenomas [3, 8]. In normal cells, Wnt is responsible for a plethora of processes including embryonic development, cell proliferation, cell polarity, specification of cell fate. The main element of Wnt pathway is β-catenin, which interacts to TCF/LEF transcription factors and activates proliferative and pro-survival transcriptional programs (more than 1000 genes including Myc). In cytoplasm, β-catenin forms complex with GSK3β/Axin/APC. This prevents translocation of β-catenin to the nucleus and promotes its proteasomal degradation. Wnt signaling leads to the disassociation of the complex and release of β-catenin.

Absence of normal function of APC tumor suppressor (adenomatous polyposis coli) results to the accumulation of β-catenin, its translocation to nucleus and subsequent induction of proliferative expressional programs. The most of sporadic colorectal adenocarcinomas (90%) harbor driver mutations within the members of Wnt pathway, with APC gene being the most frequent (70-80%) target of inactivating mutations [3, 9, 10]. In many cases, mutations of APC is the first event of the development of colorectal adenomas and subsequent carcinogenesis. In contrast, mutational activation of KRAS cannot initiate cancer *in vivo*, and only when combined with a mutation in APC mutant KRAS does promote tumor progression [11]. Mutations are not the only cause of APC deficiency. Hypermethylation of the APC promoter (18% primary colorectal carcinomas and adenomas) is alternate mechanism [4, 12].

It is well known that mutations in APC are strongly associated with CIN [3, 4]. However, dysregulation of APC—β-Catenin axis may be not obligate for the development of CIN tumors [13]. Moreover, it is unclear whether loss of APC function is truly the cause underlying CIN and whether CIN occurs before or after*APC* mutations. APCas initiator of chromosome instability pathway [4, 14-16]. During mitosis, APC clusters at the plus-ends of the spindle microtubules and co-localizes with the kinetochore, the attachment site of the mitotic spindle to the newly duplicated chromosomes [15, 17]. Losses or truncations of APC cause mitotic spindle defects that, upon somatic inactivation of other CIN-associated genes (e.g. spindle and cell cycle checkpoint genes, DNA repair, telomere maintenance, etc.) results in chromosomal abnormalities and aneuploidy as observed in the most of CIN-positive CRCs [17].

The main mechanisms of the development of CIN tumors beyond APC are related to the dysregulation of chromosome segregation, telomere formation and DNA repair. Genes related to the *BUB/MAD* family are involved in the cell cycle checkpoint system at the stage of separation of two identical chromosome copies during prometaphase. Activation of their protein products is a consequence of improper mitosis and leads to inhibition of the anaphase-promoting complex/cyclosome (APC/C, not to be confused with the APC tumor suppressor) and subsequent cell cycle arrest [18]. Mutations in genes that are involved in chromosome segregation are one of the main causes of CIN. Substitution mutations in *BUB1* or *BUBR1* impair the G2/M cell cycle checkpoint [19-21]. Furthermore, dominant mutations in the *BUBR1* are associated with both disturbances in cell cycle checkpoints and the gradual development of CIN in the cell line that initially did not have the chromosomal instability [22].

Another mechanism for the development of CIN is abnormality of the centrosome system, including the formation of additional microtubule-organizing centers [23]. It has been shown that the formation of extra centrosomes can initiate tumorigenesis of larval brain cells in *Drosophila* transgenic lines [24]. The amplification and increased expression of Aurora and Polo-like kinase genes *AURKA* and *PLK1* is among major causes of the centrosome abnormalities in colorectal cancer [25-27]. AURKA is involved in centrosome duplication, induction of mitosis, and spindle assembly. *AURKA* gene amplification is accompanied with the formation of extra centrosomes and inappropriate beginning of anaphase despite defective spindle formation [28]. Moreover, it was shown that AURKA participates in the pro-oncogenic Myc pathway [29].

Polo-like kinases PLK1-4 are involved in centrosome duplication, induction of mitosis, promotion of the metaphase to anaphase transition, and cell division [30]. It was shown that PLKs play a role in the activation of AURKA/B [31]. It has been found that Polo-like kinases are characterized by the increased expression levels in colorectal cancer and participate in the activation of migration and invasion of tumor cells [32, 33]. *AURKA* up-regulation is associated with chromosomal instability [34]. The suppression of both Aurora (in combination with the MEK) and PLK leads to inhibition of tumor cell proliferation [35, 36]. However, the association between *AURKA* overexpression and CRC prognosis is ambiguous [29, 37, 38].

The third major mechanism of chromosomal instability in colorectal cancer is associated with telomere dysfunction, which in turn causes impairments like a “breakage-fusion-bridge”, which often results in the amplification of oncogenes localized at telomeric regions [39, 40]. The study with *Terc-*deficient (telomerase RNA component) mice demonstrated association between telomere shortening and generation of intestinal carcinomas and microadenomas [41]. The importance of telomere dysfunction in the development of CRC in humans has been repeatedly mentioned. Many malignant colorectal tumors have both telomere shortening, which causes chromosomal instability, and their extension, which promotes unlimited cell proliferation [42-45]. Moreover, the positive correlation between telomere length and effectiveness of anti-EGFR therapy has been observed [46]. However, a significant lengthening of telomeres in colorectal cancer is associated with a higher degree of invasion and worse prognosis [47].

Finally, the last major mechanism of CIN development in CRC is related to the impairment of DNA damage response (DDR). It has been shown that haploinsufficiency of the H2AX histone, which is involved in the activation of ATM (ataxia-telangiectasia) during DNA damage, leads to genomic instability, and when combined with *TP53* may also initiate tumorigenesis [48-50]. One of the possible causes of association between chromosomal instability and DNA damage is that during mitosis DDR activation selectively stabilizes the interaction between microtubules and kinetochores through AURKA and PLK1 which may result in improper chromosome segregation. In case of inhibition of ATM and CHK2 proteins which are involved in cellular DNA damage response, such an effect was not observed [51].

In general, CIN-H can be treated as a factor of unfavorable prognosis [52]. In CIN tumors, as a rule, there is a loss of 25-30% alleles [7]. Besides, there is a bias in preferential losses/gains of specific regions between microsatellite stable (MSS) and instable (MSI) CIN tumors. In particular, MSS tumors, which comprises most of CIN-positive colorectal adenocarcinomas, tend to have deletions in 18q (about 50% tumors) [53]. This region harbors two crucial tumors suppressors - *DCC* (deleted in colorectal cancer) and *SMAD4*. SMADs are key intracellular components of TGF-β/BMP pathway, one of the most frequently inactivated ones in colorectal carcinomas (30-40% tumors) [3, 54].

TGF-β/BMP pathway is responsible for embryonic development, cell differentiation, apoptosis and other cellular processes. After binding of ligands (TGF-β, GDF, BMP or Activin) to the surface receptors, SMAD4 forms complex with active phosphorylated SMAD1/2/3/5/8 (R-SMADs), translocates to the nucleus and engage gene expression. The effect of TGF-β pathway strongly depends on the distinct context; in can either contribute or diminish tumor initiation and progression. Loss of SMAD4 plays an important role in the onset of squamous cell carcinomas of upper digestive tract, skin, adenocarcinomas of gastrointestinal tract [54]. The impact of SMAD4 deficiency on the development of CIN phenotype is not clear; *SMAD4* mutations in colorectal cancer probably occur before chromosomal instability, but after divergence of the microsatellite instability pathway [55, 56]. It is known about Wnt—TGF-β pathways crosstalk: SMAD4-mediated signaling inhibits intestinal neoplasia by decreasing expression of β-catenin [57].

Losses in 18q (including *SMAD4* and *DCC*) and reduced expression of SMAD4 are markers of worse response to fluorouracil-based CRC treatment [58]. Moreover, deletions of 18q, 8p, 4p and 15q, inactivation of *SMAD4*, *DCC* is considered as discussible factor of negative prognosis of colorectal cancer and other tumors [59-62].

## THE DISTURBANCE IN THE MISMATCH REPAIR SYSTEM, INCREASED FREQUENCY OF MUTATIONS IN VARIOUS GENES AND MICROSATELLITE INSTABILITY

About 15% of colorectal adenocarcinomas are characterized with an increased frequency of mutations (hypermutated, HM) that often occurs against the background of high level of microsatellite instability and disturbances in the DNA repair system (mismatch repair, MMR). According to The Cancer Genome Atlas (TCGA) project data, 60% HM-tumors are characterized by epigenetic inactivation of the *MLH1* gene, a high frequency of the *BRAF* V600E mutation and a low frequency of mutations in *APC* and *KRAS* genes. The other 40% HM-tumors, for which methylation of *MLH1* is not revealed, contain a significantly greater number of mutations and can be referred to a subtype with an ultra-high number of mutations (ultramutated, UM). UM tumors are characterized by an increased frequency of mutations in *APC* and *KRAS* genes and a reduced frequency of activating mutation *BRAF* V600E [63]. Adenocarcinomas containing mutations in genes of polymerases δ and ε (usually, against the background of microsatellite stability) can be related to UM subtype [64].

Tumors related to HM and UM subtypes develop in fundamentally different ways than most other colorectal adenocarcinomas [63]. HM subtype tumors can be a consequence of germinal mutations in one of several genes (*MSH2*, *MSH3*, *MSH6*, *PMS1*, and *PMS2*) in hereditary colorectal cancer. These genes are the tumor suppressors genes, which are inactivated in more than 10% of human cancers. Germinal mutations in these genes are associated with Lynch syndrome [65], which is characterized by a predisposition to the development of a number of malignant tumors, primarily nonpolyposis colorectal cancer and endometrial cancer.

The disturbance in the MMR system is the main cause of the high level of microsatellite instability (MSI-H), since microsatellites are particularly labile and can accumulate errors, which are not corrected [66]. However, MSS colorectal tumors show higher incidences of mutations in the *APC*, *KRAS* and TP53 genes. To determine the MSI status, the panel recommended by the National Cancer Institute (NCI) is often used. The panel consists of two mononucleotide repeats (BAT26 and BAT25) and three dinucleotide repeats (D5S346, D2S123, and D17S250). According to the classification based on the results that are obtained with the use of this panel, MSI-H tumors have instability in two or more markers, while tumors with low level of microsatellite instability (MSI-L) and MSS-tumors have instability in no more than one marker [67]. Additionally, MMR status can be determined by immunohistochemical analysis of proteins, which are involved in the repairing of unpaired bases, such as those encoded by *MLH1*, *MSH2*, *MSH6*, and *PMS2* genes [68].

Tumors with hypermutated phenotype are characterized by a more favorable prognosis. Microsatellite instability is considered and discussed as a possible marker of overall and disease-free survival, as well as of sensitivity to therapy with 5-fluorouracil (5-FU). Despite the fact that the results are contradictive [69-73], most of the recent studies suggest microsatellite instability as marker of good response to treatment with 5-FU in combination with other drugs, especially in the presence of large deletions in HSP110 [71-73]. The National Comprehensive Cancer Network (NCCN) recommends 5-FU either as monotherapy or in combination with other drugs for patients with stage III and IV colorectal cancer and stage II, if it is associated with negative prognosis. However, several research groups do not recommend using this marker whereas the other groups suggest determining the MSI status for all patients with stage II CRC, because MSI-H patients have a favorable prognosis and 5-FU therapy may be not required [74-76].

Generally, the presence of microsatellite instability in CRC is a positive prognostic factor, especially in the absence of early onset of the disease and mutations in *BRAF* gene. Against the background of an inactivated *MGMT* gene, MSI is associated with potential tumor cell resistance to methylating agents (temozolomide, dacarbazine, procarbazine), and potential sensitivity to ethylating agents (nitrosoureas).

## CPG ISLAND METHYLATOR PHENOTYPE

The existence of a group of tumors characterized with a large number of simultaneously methylated CpG islands, which results in the inactivation of several key tumor suppressor genes, was first shown for colorectal cancer. This phenomena was called CpG Island Methylator Phenotype (CIMP) [77]. CIMP-positive CRCs have their own precursor lesions, serrated adenomas, distinct from conventional adenomas, which progress and transform into CIMP-negative CRCs [78]. Based on the genome-wide data on the density of CpG island hypermethylation, three groups of tumors are mostly defined: high degree of hypermethylation (CIPM-H), low (CIMP-L) and CIMP-negative (CIMP-N) tumors [79, 80]. It was shown that CIMP-H is highly associated with hypermethylation of the *MLH1* gene and MSI-H phenotype [81, 82]. Associations of CIMP-H with proximal tumor localization, older age, female gender, high degree of differentiation and mucinous histological type, mutations in *KRAS* and *BRAF* genes and wild-type *TP53* were also identified [77, 83-85].

The description of CIMP criteria is not unified among studies. The major challenge is the selection of specific loci, methylation status of which should be used to define CIMP. Most studies use the classic panel: *MLH1, p16, MINT1, MINT2,* and *MINT31* [84, 86, 87]. This panel can be extended to include *CACNA1G, CRABP1, IGF2, NEUROG1, RUNX3, SOCS1, HIC1, IGFBP3*, and *WRN* [86, 88]. Most of the CIMP tumors are characterized by microsatellite instability and lack of chromosomal instability [3]. The accurate cause of dense aberrant DNA methylation in CIMP tumors remains incompletely clear. However, several factors that may be involved in this process were found. For example, increased expression of DNA methyltransferase-3B (DNMT3B) was detected in CIMP-H tumors [89]. *DNMT3B* expression was also increased during tumor progression and correlated with methylation level of CIMP-associated genes (*NEUROG1*, *CACNA1G,* and *CDKN2A*) and *SFRP2* gene, suggesting the presence of a certain relationship between these processes [90]. The interconnection of genetic factors, dietary features and bad habits with CIMP-H has been also analyzed in a number of epidemiological studies. Smoking was shown to be associated with the presence of CIMP-H colorectal tumors and *BRAF* mutations [91, 92]. There is a relationship between the presence of a number of genetic variants in the genes of folate metabolism (*MTHFR*, *MTR*, and *MTRR*). In particular, high probability of CIMP-H occurrence is described for patients with specific alleles of *MTHFR* gene, low consumption of folate and methionine, and high alcohol consumption [93-95].

The effect of CIMP status on prognosis has been analyzed in a large number of studies, but the results are not self-consistent enough. The results of the systematic review across 36 studies (2003—2015) suggest a poorer prognosis for patients with CIMP-positive/CIMP-H colorectal tumors compared to CIMP-N or CIMP-L CRCs [96]. The prognosis strongly depends on MSI tumor status. CIMP-H seems to be a marker of poor prognosis, but its effect can be compensated in tumors with microsatellite instability: several studies report that shortercancer-specific survival for CIMP-H group compared to CIMP-negative patients is observed only in the MSS subgroup [97, 98]. However, CIMP+/MSI+ tumors were closely associated with poorer differentiation and worse overall survival of patients compared to CIMP-/MSI+ cancers [99].

Other clinical factors or genetic characteristics can affect the significance of DNA methylation profiles as a prognostic marker. Proximally localized CIMP-H tumors are characterized with a higher rate of recurrence than CIMP-L and CIMP-N, while for the distal tumors such association was not revealed [100]. In another study, it was shown that the CIMP-H phenotype is associated with poor prognosis only in the case of rectal cancer (Asian population) [101]. Several clinical characteristics that correlate with CIMP-H status come from the presence of microsatellite instability, since CIMP-HMSI-H [86]. This makes a challenge to extract CIMP features which are not caused by MSI. However, some conclusions can be made here: CIMP is associated with *BRAF*(and less frequently - *KRAS*) mutations [3, 102-104], and some authors just ascribe poor clinical outcome of CIMP-H patients to the presence of *BRAF/KRAS*mutations [102, 105].

The effectiveness of 5-fluorouracil therapy for patients with different CIMP status was also investigated in several studies, but the data are extremely contradictory, and that can be explained by methodological differences, primarily in gene panels that were used for the status determination [70, 106-108].

## EXCISION REPAIR SYSTEM

One of the most common drugs used in colorectal cancer chemotherapy is oxaliplatin, which is a third-generation platinum derivative containing 1,2-diaminocyclohexane in its structure. In combination with 5-FU, oxaliplatin has been approved as first-line therapy for metastatic CRC [109, 110]. Like many other platinum-based compounds, oxaliplatin reacts with DNA and forms inter- and intra-strand crosslinks that block DNA synthesis and further replication. This drug has a wide spectrum of cytotoxicity *in vitro* and antitumor activity *in vivo* in different tumor models. Apoptosis of cancer cells can be caused by formation of DNA lesions, block of DNA synthesis, inhibition of RNA synthesis, and triggering of immune reactions [111]. The main role in repairing platinum-induced DNA damage is played by nucleotide excision repair (NER) system. One of the key DNA repair enzymes is ERCC1 (Excision repair cross-complementing group 1) whereas ERCC2 and XRCC1 (X-Ray repair complementing defective repair) are also of paramount importance.

It has been demonstrated that overexpression of *ERCC1* truly correlates with low sensitivity of tumors to the platinum-based drugs. Among polymorphic germinal variants of *ERCC1*, synonymous variants in 118^th^ codon (Asn118Asn) are mostly studied in the context of response to oxaliplatin therapy. This synonymic substitution is associated with the altered gene expression at mRNA and protein levels, and, as a consequence, altered NER efficacy and response to oxaliplatin [112-114]. The frequency of *ERCC1* allele, which is associated with reduced gene expression and better response to chemotherapy, is about 35% in the European population [114]. The same observations have been marked for the non-synonymous variants in *ERCC2* (Lys751Gln) [115] and *XRCC1* (Arg399Gln) genes [116].

However, the results are poorly consistent between studies. Meta-analysis of 22 studies (2004—2013; 2846 patients) revealed no differences of objective response to oxaliplatin-based chemotherapy between patients bearing distinct *ERCC1* variants, but did revealed significant differences between progression-free and overall survival. Also the impact of ethnicity factor had been demonstrated [117].

Numerous studies have shown a relationship between *ERCC1* overexpression and resistance of various other tumors to platinum-based drugs [118, 119].. In human ovarian and gastric cancers high expression level of *ERCC1* is observed in a moderate number of cases, but in colon, lung and breast cancers, the high level of the expression is observed for more than one third of patients. This may suggest the low effect of platinum-based drugs for such type of patients making this kind of therapy not only unreasonable but even dangerous because of the high toxicity of the drugs.

## MUTATIONS IN POLYMERASE GENES

Among mutations in genes encoding for different polymerases, mutations in the polymerase ε gene (Pol ε/*POLE*) are the mostly studied in colorectal cancer. Polymerase ε is one of three polymerases (α, δ, and ε) that mostly responsible for replication of nuclear DNA. Moreover, Pol ε performs the synthesis stage in the DNA repairing process and takes part in the recombination [120]. The significance of *POLE* mutations in carcinogenesis was confirmed by several studies, mainly in endometrial and colorectal cancers [64, 121, 122]. Although there is only a weak correlation between the presence of mutations in the exonuclease domain of Pol ε and the decrease of overall survival in MSS colorectal tumors [123], it was shown that *POLE* germinal mutations may be responsible for predisposition to colorectal and other cancers, including Lynch syndrome [122, 124-126]. In endometrial tumors, *POLE* mutations can play role in the development of microsatellite instability [127].

Polymerase δ also takes part in DNA reparation. It has been shown that germinal mutations in the polymerase δ gene, as well as in the polymerase ε gene, result in a high risk of development of multiple colorectal adenomas and adenocarcinomas [125]. Tumors that are formed as a result of mutations in the polymerase δ and ε genes, are characterized by an extremely high mutation frequency (over 1 million in a genome) against the background of microsatellite stability. Tumors with such characteristics may be isolated into a separate group [64]. In contrast, mutations in the polymerase α gene are rare and thus can be exposed to a negative selection in tumors [128].

## SIGNAL TRANSDUCERS (KRAS, NRAS, BRAF, PIK3CA)

*KRAS, NRAS, BRAF,* and *PIK3CA* genes belong to proto-oncogenes and encode proteins that are involved in intracellular signal transduction from growth factor receptors. Driver mutations are frequently found in these genes; they are mostly localized in the 2^nd^ exon of *KRAS* (codons 12 and 13), 3^rd^ exon of *NRAS* (codon 61), 15^th^ exon of *BRAF* (codon 600), and 9^th^, 20^th^ exons of *PIK3CA*. In normal cells, RAS proteins are activated after binding growth factors to receptor tyrosine kinases. The presence of *KRAS* (Kirsten Rat Sarcoma Viral Oncogene Homolog) mutations described above results in constitutively active KRAS protein and eliminates the need for EGFR ligand for activation downstream MAPK and PI3K/mTOR pathways. These two pathways are responsible for cell growth, proliferation, differentiation, migration in normal cells and strongly contribute to progression of tumors. BRAF (v-Raf murine sarcoma viral oncogene homolog B) is the direct downstream target of KRAS, the second element of RAS/RAF/MEK/MAPK cascade. Mutations in 600^th^ *BRAF* codon result in constitutively active MAPK signaling. Unlike KRAS, BRAF does not regulate PI3K/mTOR pathway.

Mutation status of *KRAS, NRAS, BRAF* and *PIK3CA* genes can be used as a prognostic and especially as predictive marker: the presence of a mutation in the hotspots of any of these genes is associated with a potential resistance to inhibitors of receptor tyrosine kinases, as well as to monotherapy with mTOR inhibitors [129-131]. Moreover, detection and quantification of mutated alleles in circulating tumor cells (CTC) enables non-invasive monitoring of response to therapy [132].

### *KRAS* and *NRAS* mutations

The RAS subfamily consists of about 30 structurally related proteins, small GTPases, mainly regulating cell proliferation [133]. However, only three members, KRAS, NRAS and HRAS, are of paramount importance in the context of cancer development and clinical significance. Mutations of these gene have been identified in many tumor types. In solid tumors, such as colorectal, lung and pancreatic cancers, mutations in *KRAS* gene occur much more frequently than in *NRAS* gene. The inverse picture has been observed in some hematological diseases such as acute lymphoblastic and chronic myelomonocytic leukemia and Hodgkin's lymphoma [134]. Approximately 90% of *KRAS* activating mutations are located in codons 12 and 13, which belong to the second exon. Another driver mutations are located in codons 59, 61 (third exon), 117, 146 (fourth exon) [135-137]. 30%-50% of colorectal cancers have *KRAS* mutations [3, 138]. *HRAS* mutations are not significant in CRC.

In a massive study involving patients with stage II and III colon cancer, it was shown that *KRAS* mutations do not have predictive value in assessing the overall and relapse-free survivals [139]. However, a number of studies have suggested clinical significance of *KRAS* mutations as prognostic markers [131, 140]. The presence of activating mutations in *KRAS* and *NRAS* (as well as inactivating mutations in *PTEN*) is marker of insensitivity to anti-*EGFR* therapy, particularly, using antibodies (cetuximab, panitumumab) [141, 142]. Absence of mutations in *KRAS* and *NRAS* does not imply the sensitivity to the therapy, but their presence can exactly predict the lack of response.combining mTORand EGFR inhibition as well as simultaneous inhibition of mTORand Bcl-2/Bcl-xL may improve therapeutic outcome in patients with*KRAS*- or *BRAF*-mutant CRC [143, 144].

It appears that there are other mutations associated with the resistance to cetuximab and panitumumab. For example, it has been reported that mutations in the *PIK3CA* and *BRAF* genes may are also predictive markers of absence of objective response to the anti-EGFR therapy, but conclusive demonstration on a large set of patients is currently absent. Treatment with cetuximab and panitumumab should be carried out only after obtaining data on the mutation status of the three exons of *KRAS* and *NRAS* genes, as this allows to prevent undesirable toxic effects of the drugs in the absence of objective response to the therapy.

According to the results of preclinical studies, mutations in *NRAS,* *KRAS, BRAF* genes are associated with sensitivity of tumors to inhibitors of Hsp90 and combinations of 1) sorafenib and irinotecan (in heavily pretreated mCRC); 2) MEK and PI3K/mTOR inhibitors; 3) sorafenib or MEK inhibitors; 4) Bcl-2/Bcl-xL and mTOR inhibitors; 5) anti-EGFR drugs and mTOR inhibitors [143-147]. Mutations in the *KRAS* and *NRAS* genes are actively studied as markers of response to anti-VEGF therapy (bevacizumab) [148-151].

### *BRAF* mutations

BRAF is one of three members of the RAF (Rapidly Accelerated Fibrosarcoma) serine/threonine protein kinase family. BRAF is a downstream target of KRAS. In normal cells, RAF activation occurs as a result of many complex processes, which needs binding of proteins and ligands, conformational changes, and numerous (de)phosphorylation events having regulatory character [152, 153]. *BRAF* mutations in 600^th^ codon lead to the constitutive activation of the BRAF protein and downstream elements of MAPK cascade. *BRAF* mutations are found in different types of cancer, especially in those with poor prognosis, for example, in more than 60% of metastatic melanoma, 40-70% of papillary thyroid cancer, and up to 18% of CRC [154]. Various studies have demonstrated different frequency of *BRAF* mutation occurrence in CRC - from 4% to 18% [155-157]. To date, more than 50 different *BRAF* mutations have been documented for CRC. However, in 80% of cases, mutations are represented by V600E substitution [156]. *BRAF* mutations are found highly enriched in right-sided proximal tumors [158].

*BRAF* mutations are considered to be oncogenic drivers, since they occur at early stages of carcinogenesis, causing transformation of epithelia into serrated adenomas [159]. BRAF plays an important role in CRC progression and metastasis. Particularly, BRAF constitutive activation was shown to induce disturbance of the polarity of epithelial cells *via* activating the expression of *Myc* [160].

*BRAF* mutations frequently occur in microsatellite instable tumors, which are usually associated with a failure of a mismatch repair system. According to the results of several studies, this group of patients have a favorable prognosis, and presence of *BRAF* mutations in MSI-H tumors do not correlate with reduced overall or disease-free survival [139]. *BRAF* mutations are rarely found in MSS or MSI-L tumors, which are characterized by poor prognosis [139, 161-163]. Large-scale study involving 1404 patients with stage II and III CRC demonstrated the association of *BRAF* mutations with the female gender, localization of tumor in the right side, older age (60 years or more), a high grade of anaplasia, and microsatellite instability. At the same time, *BRAF* mutations do not have prognostic potential for the assessment of time to disease progression, but can represent a marker of overall survival of MSI-L and MSS patients [139]. However, some authors assess prognostic significance of *BRAF* mutations not taking into account MSI status. The results of these studies suggest that *BRAF* mutations may be considered as an independent prognostic factor for disease-free and overall survivals in locally advanced and recurrent CRC: presence of *BRAF* mutations is associated with metastasis and represents a marker of unfavorable prognosis [157, 161, 164, 165].

In contract to *KRAS*, the predictive value of mutated *BRAF* in the context of response to anti-EGFR therapy is still discussible [159]. However, the results of two recent meta-analyses support the necessity of *BRAF* mutation study before initiation of treatment with anti-EGFR therapy. The first study by Pietrantonio *et al* included 463 RAS-wt/BRAF-mut patients from 10 trials and demonstrated that the addition of cetuximab and panitumumab treatment for the BRAF-mut patients did not improve progression free, overall survival, overall response rates [166]. The second study by Roland *et al* covered 8 trials with 3168 RAS-wt participants including 351 RAS-wt/BRAF-mut patients [167]. The results of this meta-analysis demonstrated increased both progression-free and overall survival in RAS-wt/BRAF-wt patients compared to RAS-wt/BRAF-mut (20-40% decreased hazard ratio). However, the results did not meet statistical significance criteria [167].

“Classical” *BRAF* mutations (substitutions in codons 600 and 601) are associated with tumor cell sensitivity to BRAF inhibitors: most commonly used of them are vemurafenib and dabrafenib, whereas other are also known: encorafenib, and experimental XL281 (Exelixis), CEP-32496. However, BRAF-targeted therapy may eventually fail because of the developing resistance. The vast majority of these escape mechanisms are driven by feedback reactivation of EGFR that activates in turn MAPK *via* other RAFs (CRAF) and RAS [159, 168, 169]. This suggest usage of anti-BRAF therapy in combination with inhibitors of EGFR and MEK, but here we also expect pitfalls as an acquired resistance driven by alterations in MAPK participants, which may be overcome *via* ERK inhibition [170]. “Non-classical” *BRAF* mutations may be responsible for tumor cell resistance to vemurafenib and dabrafenib, but increased sensitivity to sorafenib (multi-kinase inhibitor) and MEK inhibitors. Most likely, “non-classical” *BRAF* mutations are markers of favorable prognosis [171].

### *PIK3CA* mutations

PIK3CA is a catalytic subunit of phosphatidylinositol 3 kinase (PIK3). Mutations of *PIK3CA* gene are frequently found in various solid tumors, particularly in CRC. According to the results of several studies, *PIK3CA* mutations are present in 10-20% of CRC cases [172-175]. *PIK3CA* mutations frequently coexist with *RAS* and *BRAF* mutations in patients with advanced cancers [176]. Presence of *PIK3CA* mutations is associated with the mucinous CRC phenotype [174, 177]. About 80% of *PIK3CA* mutations are located in hotspots of 9^th^ exon (codons 542 and 545) and 20^th^ exon (codon 1047) [178]. Mutations in the 9^th^ exon are found more frequently, whereas simultaneous mutations in both 9^th^ and 20^th^ exons are very rare [177, 179, 180]. There is a gradual decrease in frequency of *PIK3CA* mutations as it moves from the proximal (cecum, 21-25%) to distal (sigmoid colon, rectum, 8-9%) sites of the intestine [177, 179]. Against the background of absence of *RAS* mutations, *PIK3CA* substitutions in exon 20 seem to be marker of poor prognosis and potential inefficacy of anti-EGFR therapy [181-183].

Surprisingly, *PIK3CA* mutations as well as overexpression of cyclooxygenase COX-2 represent a marker of good response to therapy with Aspirin [172, 184-186]. The results of meta-analysis by Li *et al* across 7 studies (35 thousand patients) suggest that post-diagnosis aspirin therapy improves CRC overall survival, especially for patients with tumors positive for *PTGS2* (COX-2) expression and *PIK3CA* mutations [187]. Another meta-analysis by Paleari *et al* included 4589 patients and revealed 29%-reduced total mortality with Aspirin treatment of PIK3CA-mut tumors, whereas no significant improvements were found for PIK3CA[185].

The major targets of Aspirin are constitutively expressed isoform of cyclooxygenase (COX-1/PTGS1) and its inducible isoform (COX-2/PTGS2), which is expressed under cytokine, inflammatory stimuli, and some growth factors. Aspirinapproximately 150—200-fold more inhibition potency for COX-1 rather than COX-2 [188]. in the substrate binding channel and inactivates the enzyme irreversibly, whereas most of other nonsteroidal anti-inflammatory drugs do it reversibly [189]. The exact mechanisms of interplay between mutations in *PIK3CA* and aspirin are still elusive [183, 190].

**Figure 1:**
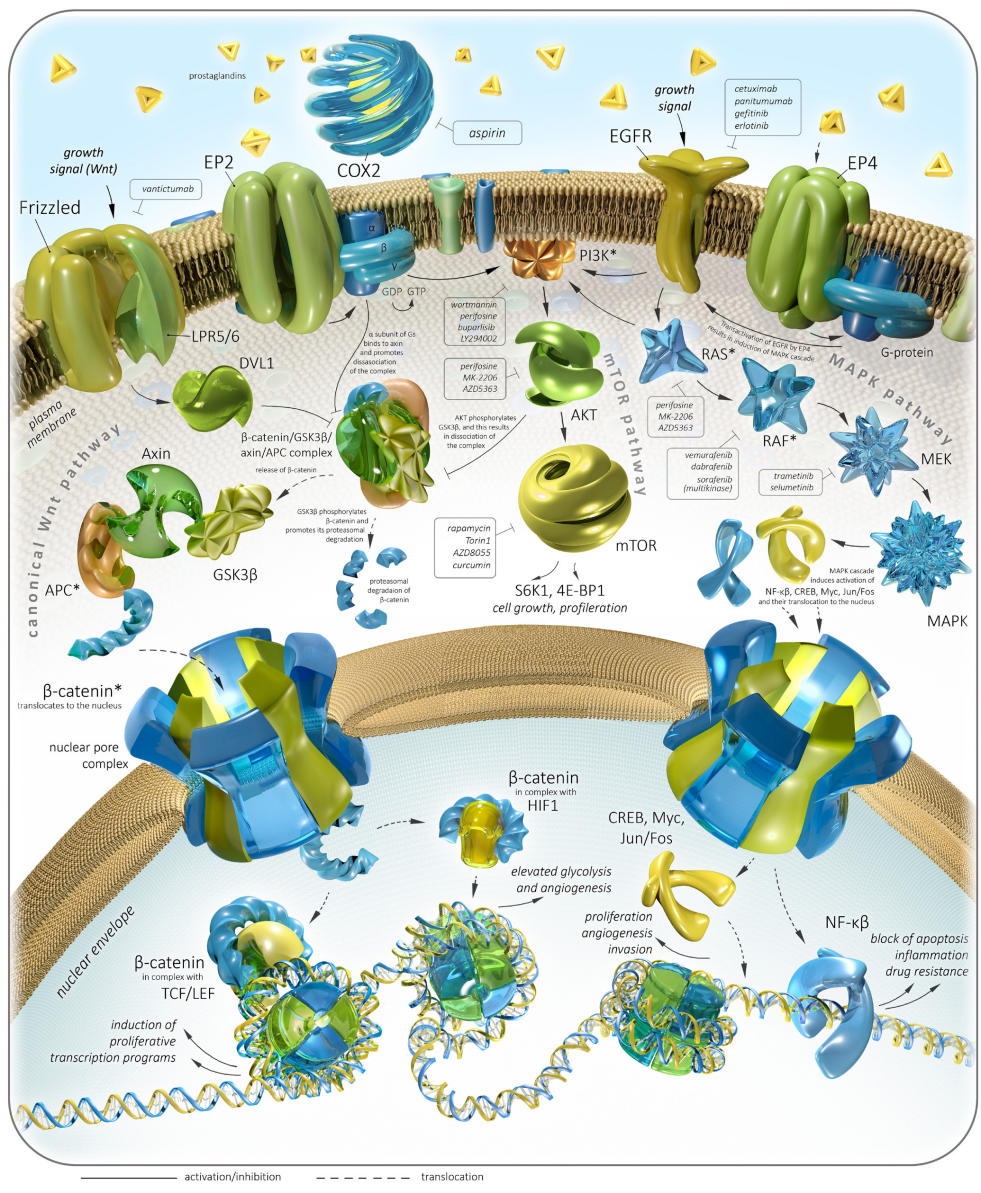
Colorectal cancer-related pathways and therapeutic targets with focus on Aspirin Asterisks (*) indicate genes harboring the most frequent and clinically significant driver mutations in CRC.

Anti-cancer activity of Aspirin may include several mechanisms. First, Aspirin contributes to the downregulating MAPK, β-catenin, Akt/mTOR, PKA, NF-kB pathways, as it was shown in different models [191-195]. Inhibition of COX-2 by Aspirin prevents synthesis of prostaglandins E2 and subsequent activation of EP1-4 prostaglandin receptors, some of which are participating colonic tumorigenesis and invasion [194, 196]. In colon cancer cells, EP2 is capable of activating β-catenin, the central element of Wnt/β-catenin pathway, which is the major player in colorectal cancer induction, growth and invasion. EP2 stimulate dissociation of β-catenin/GSK3β/Axin/APC complex and subsequent release of β-catenin by two ways: 1) α-subunit of EP2-coupling trimeric G-protein directly interacts with Axin, and 2) β/γ-subunits of G-protein activate PI3K and Akt, whereas the last one phosphorylates GSK3β [196] (Figure 1). Another prostaglandin receptor, EP4 is capable of activating MAPK pathway also by two ways: 1) EP4 induces Src—β-arrestin-mediated transactivation of EGFR, and 2) activating MAPK signaling by means of PI3K [192, 197, 198]. Pro-angiogenic activity of EP4 mediated *via* protein kinase A pathways is also known [199].

peripheral blood mononuclear cells

In platelets, COX-1 is involved in the generation of thromboxane A2 (TXA2), which promotes platelet activation and aggregation. *et al*Aspirin are not limited to interactions of tumor cells and platelets: recently it was shown that Aspirin suppresses both the growth and metastasis of osteosarcoma through the NF-κB pathway [209]. In addition, combination of tyrosine kinase inhibitor sorafenib with Aspirin may be preferred to sorafenib alone with respect to preventing metastasis [210].

Surprisingly, aspirin with combination of isoform-specific COX2 inhibitor celecoxib and lipid-lowering statin atorvastatin demonstrated more efficient inhibition of azoxymethane-induced colon carcinogenesis in rats rather than when these agents were given individually at higher doses [211].

## TP53 MUTATIONS

The *TP53* (p53) tumor suppressor plays a crucial role in the response to stress [212]. It is a component of the cell cycle checkpoint system, it sustains genome integrity, induces cell cycle arrest and apoptosis. Active TP53 is maintained at low levels in the most of cells: once activated, TP53 upregulates E3 ubiquitin-ligase MDM2, which promote TP53 proteasomal degradation [213, 214]. Activated TP53 can either induce cell cycle arrest, inhibit cell growth or promote cell apoptosis depending on different type of stress (including DNA damage) and the other context [213]. TP53 can trigger both intrinsic (mitochondrial) and extrinsic (death receptor-induced) apoptotic pathways: TP53 activates the expression of pro-apoptotic proteins Bax, Noxa and PUMA and downregulates anti-apoptotic Bcl-2. This leads to the formation of mitochondrial permeability pore and subsequent release of cytochrome c which is responsible for the induction of caspase cascade [215-217]. On the other hand, active TP53 upregulates the expression of some death receptor genes, such as Fas, DR5 and PIDD [214].Among TP53 targets there are well known tumor suppressors GADD45, WAF1 (p21), Rb, 14-3-3-σ. he role ofhas been shown to go beyond its effects on cell cycle and apoptosis [218]. TP53 is involved in the metabolic reprogramming, regulation of intracellular reactive oxygen species levels, once more highlighting its role in cell death or cell survival [219-221].

Failure of TP53 pathways is one of the hallmarks of cancer cells [222, 223]. Up to 50% of human malignancies contain *TP53* mutations. In the rest of tumors, *TP53* pathways have other deficiencies to ensure uncontrolled growth and proliferation. These alterations play an important role in CRC onset and progression and may represent a marker of the response to chemotherapy, radiotherapy or their combination. Driver mutations of most of tumor suppressor genes are associated with almost complete loss of protein function or expression. In contrast, not all of TP53 driver mutations result in complete loss of functionality. Tumor-associated mutantoften gain new tumorigenic activities. In particular, not only completely losses of activity but ‘gain-of-function’ -mutantstumor metabolic reprogramming which promotes tumor progression and invasion [220, 221, 224, 225]. TP53 mutant proteoforms may inhibit apoptosis, which normally occurs in the response to ionizing radiation and anti-cancer drugs (doxorubicin, platinum-containing agents, *etc.*) [226-228]. The presence of *TP53* Pro72Arg germlinne variant (rs1042522) may be associated with increased sensitivity to 5-fluorouracil [229].

## GERMINAL VARIATIONS IN GENES ENCODING DRUG METABOLISING ENZYMES

### Mutations of dihydropyrimidine dehydrogenase (DPYD)

The *DPYD* gene encodes a cytoplasmic enzyme involved in pyrimidine catabolism. Its expression level and enzymatic activity negatively correlates with both efficiency and toxicity of 5-fluorouracil and related antitumor drugs [230]. Germline inactivating *DPYD* mutations cause the so-called DPYD syndrome involving life-threatening complications of 5-FU or capecitabine treatment [231]. In order to prevent these potentially fatal outcomes, DPYD testing before administration of pyrimidine antimetabolites is advocated by several groups [232]. For the carriers of a non-functional *DPYD* allele, current guidelines recommend complete avoidance of 5-FU and related drugs in case of homozygous mutation and at least 50% dose reduction in case of heterozygous one [233]. *DPYD* mutation analysis may be rather cumbersome due to absence of mutation hotspots (except 3 very high risk alleles) [234], unknown significance of many allelic variants and possible involvement of epigenetic mechanisms (including but not limited to the levels of *DPYD* promoter methylation and *miR-494* expression [235]). Therefore, ELISA-based testing for DPYD enzymatic activity seems to be a reasonable alternative.

However, wide clinical acceptance of DPYD testing in general is hampered by relatively low incidence of *DPYD* mutations (minor allele frequency for each substitution is below 0.01%). In CRC (as well as in gastric cancer and several other solid tumors), high levels of DPYD expression and activity in tumor cells correlate with relative resistance to 5-FU and capecitabine [236, 237] but not to S-1 or raltitrexed [238], whereas tumors with low *DPYD*, low *TYMS* and high *TYMP* expression levels were found to be exquisitely sensitive to capecitabine [239]. These trends were seen in many retrospective analyses, but no definitive prospective studies on the subject were ever conducted, so validated algorithms for antimetabolite therapy personalization in CRC are still lacking.

### Variations in UDP glucuronosyltransferase (*UGT1A1*)

The *UGT1A1* gene encodes a cytoplasmic enzyme involved in detoxification of a wide range of metabolites and xenobiotics including bilirubin and SN-38, the most active metabolite of irinotecan. Several allelic variants in the TATA-box of the *UGT1A1* promoter (UGT1A1*28 is the most common alternative allele) are associated with a decreased level of its expression and activity, which in turn leads to rapid accumulation of its unmodified substrates in several tissues and organs, mainly in the liver and blood. Individuals harboring UGT1A1*28 allele are at high risk for transitory neonatal hyperbilirubinemia (Gilbert and Kriggler-Nayyaar syndromes) and for life-threatening toxicities of irinotecan, including deep neutropenia and fatal diarrhea. Therefore, irinotecan dose adjustment is highly required in CRC patients homozygous for this allele and may also be discussed in heterozygous cases having any other risk factors for increased sensitivity to irinotecan [240, 241].

## CLASSIFICATION OF COLORECTAL CANCER

**Figure 2:**
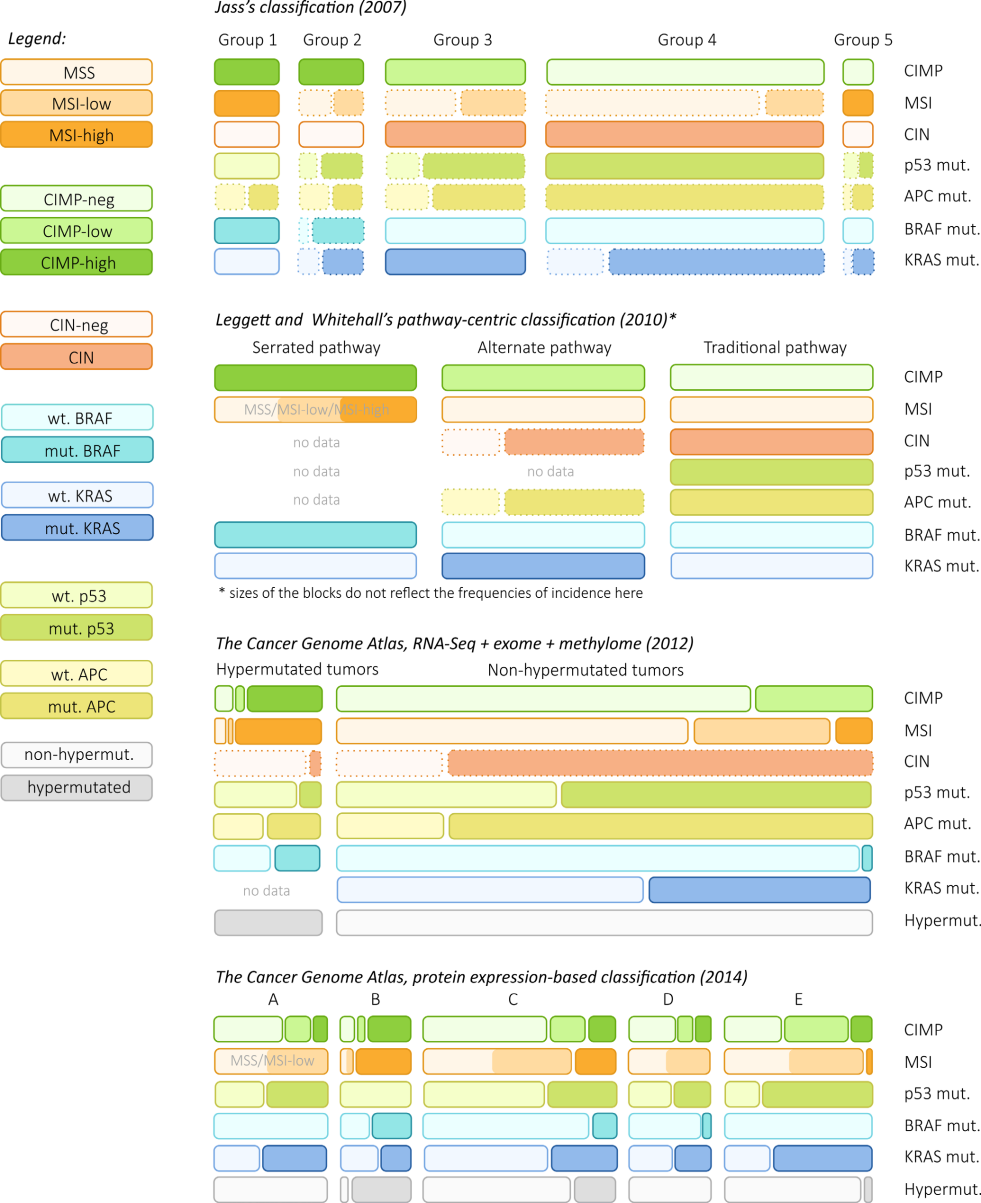
Four approaches of colorectal cancer classification Size of boxes indicates the frequency of the corresponding group or feature (except Leggett's approach). Dotted boxes indicate that the frequency of feature/group is evaluative.

A set of molecular markers is usually used for CRC classification, which should accelerate understanding of the causation and facilitate clinical management in the areas of both prevention and treatment. Traditionally, colorectal cancer has been classified according to three molecular features: chromosomal instability, microsatellite instability, and the CpG island methylator phenotype. Unfortunately, such a classification is very conditional, and sets of markers of each group do not uniquely represent their unequivocal signs. In 2007, with the development of molecular genetics, Jeremy Jass proposed to classify CRC into five molecular subtypes (Figure 2):

1. CIMP-H, MSI-H and *BRAF* mutation;
2. CIMP-H, MSI-L or MSS/*BRAF* mutation;
3. CIMP-L/MSS or MSI-L/*KRAS* mutation;
4. CIMP-negative/MSS;
5. CIMP-negative/MSI-H or Lynch syndrome.

The molecular pathways are determined at an early stage of tumor formation. Serrated polyps seem to be the precursors of CRC types 1 and 2, whereas CRC types 4 and 5 evolve through the adenoma-carcinoma transformation. CRC types 1 and 4 represent two “reference points” with minimal overlap, Each type has its own histological features and clinical picture [242].

The evolution of CRC classification tended to shift from descriptive to integrative approaches and to link of traditional CRC classification (CIMP, MSI/MSS, CIN) to the underlying alterations of signaling pathways (Wnt, TGF-β, Ras) and driver mutations. In 1990, Eric Fearon and Bert Vogelstein suggested a multistep model of colorectal carcinogenesis which became a paradigm for the next years [243]. This model describes *APC* inactivating mutations as the first event of CRC development. Then, *KRAS* activating mutation are taking place. Further, other mutations in elements of TP53, PI3K, TGF-β and other pathways occur [243, 244]. This model became a basis for understanding colorectal cancer development. During next years, the principle of the model remained unchanged: several hits are needed for CRC onset. However, the model was subject to many refinements and additions [245, 246].

In 2010, Barbara Leggett and Vicki Whitehall summarized accumulated data on CRC development and suggested new classification system of sporadic CRC based on molecular pathways of its onset and progression [5]:

1. *Serrated pathway*. The initial event is *BRAF* mutation, which leads to the formation of microvesicular hyperplastic polyp. Subsequent promoter methylation of *p16*, *IGFBP7* results in the formation of sessile serrated adenomas. Finally, methylation of either *MLH1* and TGFβ receptor II genes occurs (and this results to MSI-H, CIMP-H cancer) or other genes along with possible mutations in *TP53*, losses of 18q, deregulation of Wnt pathway (this results to MSS, CIMP-H cancer).
2. *Alternative pathway*. This pathway has two alternate starting points. The first one is *KRAS* mutations followed with possible methylation of *p16*, *IGFBP7*, deregulation of Wnt pathway. This leads to the development of traditional serrated adenomas. The second starting point is mutation in *APC* leading to the formation of small tubular adenomas. Then, *MGMT* methylation, *KRAS* mutations along with chromosomal instability occurs. This results in the onset of tubulovillous adenoma. Both tubular and tubulovillous adenomas develop into MSS, CIMP-L colorectal cancer.
3. *Traditional pathway.* Like alternate pathway, starting point here is *APC* mutation with subsequent formation of small tubular adenoma. Then, *TP53* mutations, losses of 18q and chromosomal instability take place. This results in the growth of tubular adenomas with the development of severe dysplasia. Finally, it can result in MSS, CIN-H, CIMP-negative colorectal cancer with no mutations in *BRAF* and *KRAS*.

With the spread of high-throughput methods of genome and transcriptome sequencing, several novel approaches for CRC classification have evolved. The Cancer Genome Atlas (TCGA), the greatest project in the field of molecular oncology, comprises genome, transcriptome and methylome data for several hundred CRC samples. In 2012, analysis of these multidimensional datasets (RNA-Seq, mutations, methylation) allowed for a new look at the classification of colorectal cancer [3]. Two major classes of CRC were found: hypermutated (HM, > 10 non-silent substitutions per 1 Mb) and non-hypermutated (non-HM) tumors (Figure 2). These groups demonstrated the most dramatic difference in gene expression profiles between each other. Intra-group variation was significantly lower than inter-group. In turn, each group could be subdivided into several subgroups according to alterations in the signaling pathways (Wnt, TGF-β, RTK/RAS, PI3K, TP53), driver mutations and classical subtyping (CIMP, CIN, MSI). HM and non-HM tumors revealed the following differences in Wnt, TGF-β and RAS - three pathways which are the mostly altered in CRC:

- TGF-β signaling was repressed in 84% and only 27% of HM and non-HM tumors, accordingly;
- 80% and 59% of HM and non-HM tumors demonstrated activation of RTK/RAS signaling;
- 97% HM tumors demonstrated activation of Wnt pathway as opposed to only 92% of non-HM tumors;

Non-HM tumors have more dramatic changes in gene expression profiles. The following regularities can be remarked here:

- down-regulation of FOXA1 targets and genes involved in inflammatory response (only non-HM tumors);
- up-regulation of integrins and genes responsible for angiogenesis (only non-HM);
- increased expression of MYB targets in HM tumors; increased expression of p63 targets and non-homologous end joining repair system genes in HM tumors.

The further subdivision of CRC into groups smaller than HM and non-HM is strongly complicated because of the presence of various combinations of pathway alterations. However, some observations can be done here. For example, non-HM tumors with repressed TGF-β pathway have higher frequency of RTK/RAS activation than non-HM tumors with quasi-intact TGF-β pathway. Among the 30 HM tumor samples, 23 (77%) were MSI-H but none of non-HM tumors had MSI-H phenotype. Moreover, 19 of these MSI-H 23 tumors had *MLH1* methylation, 17 of 23 had CIMP-H phenotype. Remarkably, excluding hypermutated samples, colon and rectum cancers demonstrated similar patterns of genomic alterations [3].

One year earlier, in 2011, Anita Sveen *et al* evaluated frequencies of exon skipping or inclusion in 160 colorectal cancer samples using microarrays and identifies transcriptome instability (TIN) as one of the CRC characteristics [247]. TIN tumors show skewed exon usage profiles, which strongly correlated with the expression of almost half of 280 splicing factors. Tumors of TIN phenotype account for 30-55% colorectal cancers. There were significant associations between transcriptome instability and reduced patient survival. However, no association between MSI and TIN was found. In 2014, Sveen extended this conception to other cancer types and identified TIN as pan-cancer characteristics [248].

Another approach for CRC classification has emerged from the results of CRC proteomic study in 2014 [2]. Protein abundances in 79 TCGA colorectal cancer samples were analyzed using panoramic mass-spectrometry approaches (LC-MS/MS). Cluster analysis of these data revealed five groups of samples with the distinct patterns of protein expression. These subtypes (A-E) contained 15, 9, 25, 11, and 19 samples, respectively. It was also shown that the mRNA level does not always reliably predict protein abundance. 32% of genes did not have statistically significant correlation between mRNA and protein levels. According to the results of gene set enrichment analysis based on Gene Ontology data, biological functions of proteins that have consistent mRNA and protein levels were different from those without the consistency.

The authors studied the associations between protein expression patterns and genomic and epigenomic characteristics of the tumors. Below we provide a summary for each subtype:

- MSI-L or MSS, 18q losses, *KRAS* mutations, absence of *POLE* and *BRAF* mutations, CIMP-N;
- MSI-H, hypermutation, *POLE* and *BRAF* mutations, absence of *TP53* mutations and 18q losses, CIMP-H;
- medium rate of *TP53* mutations, several samples with MSI-H, *BRAF* and *POLE* mutations, rare CIMP; this is the largest and heterogeneous group;
- MSI-L or MSS, 18q losses, *TP53* mutations, rare CIMP;
- “invasive” TCGA subtype; MSI-L or MSS, ubiquitous *TP53* mutations and 18q losses, rare CIMP, *HNF4α* amplification and overexpression.

Finally, in 2013 Enric Domingo *et al* analyzed somatic mutations in 906 specimens (stages II and III) taken from patients participating VICTOR clinical trial and found many associations, both novel and well-known (e.g. co-incidence of CIN and mutations of *TP53*; MSI and *BRAF* mutation). Based on this data, Domingo *et al* suggested alternate system for CRC classification which assumes division into seven groups:

1. MSI and/or *BRAF* mutations;
2. CIN and/or *TP53* mutations, wild-type *KRAS* and *PIK3CA*;
3. *KRAS* and/or *PIK3CA* mutations, CIN, wild-type *TP53*;
4. *KRAS* and/or *PIK3CA* mutations, CIN-negative, wild-type *TP53*;
5. *NRAS* mutations;
6. no mutations;
7. others.

Such complexity of CRC classification reflects the diversity of possible scenarios of CRC development. These pathways not only have different starting points but also a number of bifurcation points during the disease progression from polyps, adenomas to adenocarcinomas. Future investigations are needed to construct the unified and exhaustive CRC molecular classification that can finally be translated into clinical practice. In conclusion, below we summarize the data regarding markers of prognosis, efficacy of treatment and side effects of chemotherapy.:

- Markers of unfavorable prognosis: CIN; deletions in 18q (discussable), 8p, 4p, 15q regions and inactivation of *SMAD4*, *DCC,* lengthening of telomeres in CIN tumors; CIMP-H in combination with MSS; *BRAF* mutations in MSS or MSI-L tumors; mutations in *PIK3CA* (20^th^ exon)*, KRAS, NRAS.*
- Markers of favorable prognosis: hypermutated tumor phenotype; MSI-H, especially in the absence of early onset of the disease and mutations in *BRAF* gene; CIMP-H in combination with MSI; proximal localization of CIMP-H tumors.
- Mutations in *KRAS*, *NRAS*, *BRAF*, *PIK3CA* (20^th^ exon) are associated with resistance to targeted anti-EGFR therapy (cetuximab, panitumumab): .
- Mutations in *KRAS, NRAS,* *BRAF* are potential markers of sensitivity to inhibitors of Hsp90, combinations of 1) sorafenib and irinotecan (in heavily pretreated mCRC), 2) MEK and PI3K/mTOR inhibitors, 3) sorafenib or MEK inhibitors, 4) Bcl-2/Bcl-xL and mTOR inhibitors, 5) anti-EGFR drugs and mTOR inhibitors.
- MSI-H is associated with sensitivity to 5-FU and other fluoropyrimidines.
- Loss of 18q (including *SMAD4* and *DCC*), reduced expression of *SMAD4* is associated with lower response to 5-FU.
- Low expression level and specific allele variants of *ERCC1*, *ERCC2*, *XRCC1* are associated with sensitivity of tumors to the platinum-based drugs.
- *PIK3CA* mutations and COX-2 overexpression are associated with longer PFS and OS in patients receiving adjuvant therapy with COX inhibitors including aspirin.
- *TP53* Pro72Arg germlinne variant (rs1042522) may be associated with increased sensitivity to 5-FU.
- Expression level and specific allele variants of *DPYD* may seriously influence both efficacy and toxicity of 5-FU, capecitabine.
- Several germline variants in *UGT1A1* are associated with severe toxicities of irinotecan.
